# Strategies to Raise Awareness About Screening and Vaccination for the Human Papillomavirus Among Women in Limpopo Province, South Africa

**DOI:** 10.3390/ijerph23050657

**Published:** 2026-05-15

**Authors:** Matodzi Pertunia Mushasha, Lebitsi Maud Modiba

**Affiliations:** 1 Department of Health Studies, College of Human Sciences, University of South Africa, Pretoria 0003, South Africa; 2 Department of Public Health Pharmacy and Management, School of Pharmacy, Sefako Makgatho Health Science University, Pretoria 0204, South Africa; lebitsimodiba@icloud.com

**Keywords:** human papillomavirus, HPV screening awareness, HPV screening uptake, women’s health, South Africa

## Abstract

**Highlights:**

**Public health relevance—How does this work relate to a public health issue?**

- Low awareness of human papillomavirus (HPV) screening and vaccination contributes to delayed detection and increased risk of cervical cancer among women.
- Identifying effective awareness strategies is essential to improve knowledge and promote preventive health behaviours.

**Public health significance—Why is this work of significance to public health?**

- Cervical cancer remains an important public health concern in South Africa, yet many cases can be prevented through HPV screening and vaccination.
- Understanding community perceptions and awareness gaps can guide targeted interventions to improve the adoption of HPV prevention services.

**Public health implications—What are the key implications or messages for practitioners, policy makers and/or researchers in public health?**

- Public health professionals should implement culturally appropriate education programmes to increase the awareness and acceptance of HPV screening and vaccination.
- Policymakers and researchers should strengthen community awareness campaigns and integrate HPV education into existing primary healthcare services.

**Abstract:**

Background: Human papillomavirus (HPV) is a serious infection which is primarily transmitted through sexual intercourse. Almost 100% of cervical cancers are caused by HPV. Limited awareness of HPV leads to delayed cancer diagnoses, significantly increasing mortality and morbidity rates. Aim: The purpose of this study was to develop strategies to increase awareness of human papillomavirus screening and vaccination among women in Limpopo Province, South Africa. Setting: This study was carried out in the Vhembe District of the Thulamela Municipality of Limpopo Province. Methods: The E-Delphi method was used, and the researcher used a quantitative approach. A total population of 15 nursing managers was part of the study. Questionnaires were used to collect data. Data were analysed using the statistical package for the social sciences version 26. Results: In Round 1, 8 (53.3%) of the 15 participants strongly supported the strategy of updating women with the most recently revised HPV screening guidelines. In Round 2, consensus was achieved, with 14 (93.3%) of the participants strongly agreeing that the development of teaching programmes in healthcare facilities is necessary. This indicates a strong convergence of expert opinion on the importance of structured educational interventions to support the implementation of the strategy. The consensus in this study was defined as ≥70% agreement between participants on each item. Conclusions: The lack of awareness of HPV is concerning because early detection and treatment can prevent serious health problems. The study used the E-Delphi method to assess the effectiveness of strategies to increase awareness of HPV screening and vaccination in women. Contribution: Health policy initiatives may improve public awareness of HPV and vaccination, especially by focusing on educating nurses, which could improve women’s awareness and encourage HPV screening and vaccination.

## 1. Introduction

HPV is the virus that is responsible for causing diseases such as cervical cancer, which is the fourth most prevalent cancer among women worldwide. Furthermore, 76% of cervical cancer worldwide is caused by HPV types 16 and 18 [1]. Nearly 100% of cervical cancer cases are caused by HPV, regardless of the specific types of HPV involved. In 2020, approximately 90% of the new incidences of HPV-related diseases occurred in low- to middle-income countries. In 2012, Africa recorded 21% new cases and 26% global deaths from HPV-related diseases [2]. In Africa, the burden of HPV infections is reported to be very high. However, there is still a low uptake of HPV vaccination. Furthermore, healthcare providers are the main source of information for patients with respect to HPV and vaccination, but patients are still not aware of HPV screening and vaccination [3].

The lack of knowledge about HPV vaccination and screening in developing countries such as Nigeria is the leading barrier to the prevention of HPV infection [4]. HPV vaccination and screening play important roles in reducing the high mortality and morbidity rates of HPV-caused cancers. Active promotion of HPV vaccination and detection is required to improve the high mortality and morbidity rate [1]. Developed countries such as Australia have achieved a high coverage of HPV vaccination, reported at approximately 80% among females and 76% among males based on 2019 data. This high uptake of vaccination has contributed to a significant decline in HPV infection rates. Furthermore, Australia reports one of the lowest cervical cancer burdens worldwide, with incidence rates of approximately 6–7 cases per 100,000 women and mortality rates of approximately 1.7–2.0 deaths per 100,000 women. These rates have declined substantially over time, reflecting the impact of effective HPV vaccination and organised screening programmes. These achievements highlight the effectiveness of comprehensive HPV prevention strategies, including vaccination and early detection interventions. In developing countries such as Kenya, the uptake of HPV vaccination is very low; this was observed in the first dose of HPV vaccination, with coverage rates of 33%, and the second dose declined to 16% [2].

It appears that HPV screening coverage of the Vhembe district in Limpopo Province, South Africa, has not improved from 2017 to 2020. In 2017/2018, the coverage was at 55.5%, and decreased to 54.4% in 2018/2019; in 2019/2020, the coverage dropped even further to 31.6%. This is evidence that awareness of HPV vaccination and screening is still required to increase vaccination uptake and reduce the high incidence rate. The Health Belief Model (HBM) provides a useful behavioural framework for understanding the uptake of HPV vaccination and screening services. The model states that individuals are more likely to engage in preventive health behaviours when they perceive themselves as susceptible to a disease, believe that the disease has serious consequences, and perceive clear benefits from taking action, while also experiencing minimal barriers to action. In the context of HPV prevention, women’s acceptance of screening and vaccination is influenced by their perceived susceptibility to HPV infection, the perceived severity of cervical cancer, the perceived benefits of vaccination and screening, and perceived barriers such as cultural beliefs and lack of knowledge or access. Therefore, this framework is relevant in explaining the persistent low adoption of HPV-related preventive services observed both in developing countries and in the Limpopo Province context. This study aimed to develop strategies to increase awareness of the screening and vaccination of the human papillomavirus among women in Limpopo province, South Africa.

This paper presents a set of strategies that aim to increase the awareness and adoption of HPV screening and vaccination. Although these strategies are not the primary results of the main study, they represent an important component derived from the study’s triangulation process, integrating insights from both quantitative and qualitative data. Taking advantage of these combined findings, strategies are tailored to address the specific gaps and needs identified throughout the study. Although global and national frameworks such as those of the World Health Organization and the South African National Department of Health provide broad guidance on HPV prevention, there is limited evidence on how these strategies can be effectively contextualised and implemented in rural primary healthcare settings. This study addresses this gap by developing and validating context-specific strategies to improve HPV screening and vaccination awareness among women in Limpopo Province.

## 2. Materials and Methods

This study represents a subsequent phase of a larger study that initially assessed the knowledge and awareness of HPV screening and vaccination among participants. The findings from the primary study informed the development of the strategies presented in this paper. A smaller, purposively selected sample was used in this phase to validate the strategies developed. However, it is acknowledged that the small sample size may limit the external generalisability of the findings. This study forms part of a larger research project conducted in two distinct phases: qualitative and quantitative.

### 2.1. Research Design

A quantitative research method with an E-Delphi approach was used, in which participants completed individual questionnaires independently and anonymously. The initial set of strategies was developed based on the findings of the main study, which employed a mixed methods approach incorporating both quantitative and qualitative data. Key themes and gaps identified from the empirical findings informed the generation of preliminary strategies. These strategies were then presented to the expert panel during Round 1 of the Delphi process for evaluation, refinement, and validation. Although this study followed a structured approach to collecting expert opinions, it adhered to key principles of the Delphi technique, including the use of multiple rounds, controlled feedback, and the establishment of consensus. Therefore, the study is appropriately classified as a Delphi study rather than a single-round expert opinion survey. After each round, the aggregated responses were analysed and shared with the participants in the form of controlled feedback, allowing them to review the general responses and reconsider their points of view in subsequent rounds to reach consensus.

The Delphi technique was considered appropriate for this study as it enables the systematic collection and refinement of expert opinions to achieve consensus on complex public health issues where empirical evidence may be limited or context-specific. Given that this study aimed to develop and validate strategies to improve HPV screening and vaccination awareness, the use of a panel of experienced healthcare professionals allowed the generation of informed, practice-based recommendations. Furthermore, the iterative nature of the Delphi process facilitated the refinement and prioritisation of strategies through controlled feedback. Data were systematically collected and analysed in both phases, enabling a comprehensive understanding of the topic. The strategies presented here are based on this main study, leveraging the insights gained through the integration of qualitative and quantitative findings to inform targeted recommendations.

### 2.2. Population

The population consisted of nursing managers. Purposive sampling was utilised to select participants for the study. The researcher specifically targeted participants who were well informed about the relevant topics [5]. The sample included nurses with specialities in primary health care, obstetrics, and gynaecology, trained for the detection of HPV infection. Nursing managers were primary participants due to their specialised expertise in health care management and clinical nursing, and as leaders in the field, they are suitable for validating strategies aimed at increasing awareness of HPV screening and vaccination among women.

### 2.3. Sampling Size

The study included the entire population of 15 experts drawn from 15 selected clinics. All nurses with training in HPV infection screening, primary healthcare, obstetrics, and gynaecology and with more than five years of professional experience were included/selected. Consequently, no sample size was determined, since a total population sampling approach was used.

The inclusion of 15 experts was considered sufficient for the E-Delphi process, as E-Delphi studies typically rely on small, purposively selected panels of experts rather than large samples. The emphasis is on the quality of expertise and the relevance of participants, rather than on the number of participants.

### 2.4. Inclusion Sampling Criteria

- Nursing managers trained for HPV infection detection or PHC speciality.
- Nursing managers who have specialised in gynaecology, obstetrics, and midwifery.
- Nursing managers with five years or more of work experience selected clinics.

### 2.5. Data Collection Instrument

In this study, a questionnaire was used as a data collection tool, divided into two sections. Table A1 collected demographic information from the participants and Table A2 contained questions related to the development and validation of strategies. Table A2 included various strategies to raise awareness among women about HPV screening and vaccination. Participants were provided with five Likert scale response options for each strategy: “agree”, “strongly agree”, “uncertain”, “disagree”, and “strongly disagree” (see Appendix A) The questionnaire was written in English and was expected to take approximately 20 to 30 min to complete.

In this study, the validity of the face and content was confirmed through a process involving nurses and physicians who are specialists in reproductive health and a supervisor who is a nurse, midwife, and women’s health reproductive specialist in women’s health. This process ensured that the scope was covered in a comprehensive way and that the questions were appropriately structured. Pretesting was carried out in this study before carrying out the data collection of the main study to rectify the data collection instrument to ensure that the questions were clear and understandable. The pre-test phase helped improve the quality and reliability of data collection in the main study.

### 2.6. Data Collection Procedure

The data collection procedure started after obtaining ethical clearance from the Ethics Committee of the University of South Africa. The study was approved on 29 November 2021, CREC Reference: 63279401_CREC_CHS_2021. After obtaining ethical clearance, an application was made to the provincial Department of Health to carry out the study at selected clinics in Thulamela municipality and Vhembe district, which was approved [LP-202201-021]. Prior to data collection, participants were informed about what the study entails, including voluntary participation. Furthermore, consent to participate in the study was obtained from the participants. The researcher collected the data personally. Respondents who met the inclusion criteria were invited to one of the selected clinics.

A quantitative research method was used using the E-Delphi technique. Participants completed individual questionnaires independently during two structured rounds. The responses were completed privately and anonymously, and no direct discussion was allowed during questionnaire completion to maintain the integrity of the E-Delphi process. The consensus was defined as ≥70% agreement among participants on each item. After the first round, responses were analysed using descriptive statistics and controlled feedback in the form of aggregated group results. This allowed participants to reflect on the overall responses before completing the second round. Participants were also given the opportunity to provide qualitative feedback, suggest modifications, and propose additional strategies during each round, ensuring that the process remained flexible and inclusive of expert input.

The stability of the responses was evaluated by comparing the distribution of the responses between Round 1 and Round 2, with minimal variation indicating that stability had been reached and that additional rounds were unnecessary.

### 2.7. Data Analysis

The data collected was entered into a Microsoft Excel sheet and then exported to a social statistical package for the social sciences, software version 26. Frequency analysis was used to determine the level of agreement among respondents when ranking strategies. This process allowed the researcher to adjust the strategies based on the rankings and revisit them for consensus decision-making. Once consensus was reached, it captured the broad range of opinions of the expert group. Collective responses were summarised using measures of central tendencies, standard deviation, and frequency distribution. After analysing the data, the researcher implemented strategies to raise awareness of HPV screening and vaccination among women.

## 3. Results

### 3.1. Socio-Demographic Data

The researcher used descriptive statistics to analyse demographic data, including gender, years of practice experience, and professional background, among study participants. Frequency distributions and percentages were calculated for each variable to improve the understanding of the unique characteristics of the participants. Demographic data was thoroughly analysed and discussed. Table 1 presents a summary of sociodemographic information.

#### 3.1.1. Gender

This research study included both men and women. Most of the 12 respondents (80.0%) were women, while only 3 (20.0%) were men.

#### 3.1.2. Experience in Practice

The results of the study indicated that the participants had different levels of experience in their practice, with a duration of five years or longer. A majority of seven respondents (46.7%) possessed 10–14 years of practice experience; only two (13.3%) of the respondents reported having five to nine years of practice.

#### 3.1.3. Profession

In this research study, the results indicated that nine participants (60.0%) were nursing managers who specialise in PHC speciality, while only two respondents (13.3%) had a specialisation in obstetrics and gynaecology.

### 3.2. Round One: Strategies to Raise Awareness Among Women About HPV Screening and Vaccination in Limpopo Province

The first round of the Delphi process focused on evaluating strategies to increase awareness of HPV screening and vaccination. No consensus was achieved at this stage. Therefore, a second round was conducted, which resulted in agreement among experts, indicating convergence of opinion following feedback. The E-Delphi panel comprised 15 experts involved in the development of these strategies. Table 2 presents the strategies identified in Round 1 for raising awareness among women in Limpopo Province about HPV screening and vaccination uptake.

#### 3.2.1. Information for Women on the Latest Updates in the HPV Screening Guideline

There was strong agreement among participants on the need to update women on the latest HPV screening guidelines, with 53.3% strongly agreeing, indicating a high level of support for this strategy. Similarly, the participants showed clear consensus on the implementation approach, as all respondents supported the establishment of an educational curriculum in primary healthcare settings, reflecting the recognition of structured education as essential for effective dissemination. Regarding the implementation timeline, the responses were more varied. Although a majority supported implementation within one month, they preferred a two-month timeframe. This suggests a general preference for early implementation, although with some uncertainty about feasibility. There was also a strong consensus on the responsibility for implementation, with all participants agreeing that nursing managers and facility managers should lead this strategy. This highlights the shared view that leadership at the facility level is critical for successful execution.

#### 3.2.2. Conducting HPV Awareness Campaigns in Primary, Secondary, and Tertiary Educational Institutions

There was strong overall support for launching HPV awareness campaigns in primary, secondary, and tertiary education settings, with 73.3% agreeing and 26.7% strongly supporting the strategy. This indicates a broad consensus on the importance of school- and institution-based awareness as a key intervention for HPV prevention. Similarly, there was clear agreement on the development of a structured campaign programme, with 66.7% agreeing and 33.3% strongly supporting this requirement, highlighting the perceived need for formalised planning to ensure effective implementation. In contrast, responses regarding the implementation timeline were widely distributed, with preferences spreading across one month, two months, three months and six months. This pattern suggests there is no clear consensus on timing, reflecting differing views on feasibility and readiness for implementation. However, there was strong agreement on governance, i.e., that the National Department of Health should oversee implementation. This reflects a shared perception that national-level coordination is necessary for effective rollout.

#### 3.2.3. Implementation of the HPV Programme on Community Radio and Television Channels

There was a strong consensus that HPV programmes should be implemented through community radio and television channels, with 80.0% agreeing and 20.0% strongly agreeing. This indicates a broad support for the use of mass media platforms to enhance HPV awareness at the community level. Similarly, there was clear agreement on the development of structured broadcast programmes, with 66.7% agreeing and 33.3% strongly agreeing, highlighting the recognition that formalised content is necessary for effective health communication through media channels. However, no clear consensus emerged on the implementation timeline, as responses were distributed over one month, two months, three months, and six months. This pattern reflects divergent views on readiness and feasibility, suggesting uncertainty about how quickly media-based interventions can be operationalised.

#### 3.2.4. Providing Daily Health Education at PHC on HPV Screening and Vaccination

There was a strong overall agreement on the implementation of a strategic plan to ensure daily health education on HPV vaccination and screening in primary healthcare facilities, with 73.3% agreeing and 26.7% strongly supporting this initiative. This reflects a shared recognition of the importance of continuous health education in improving HPV awareness and prevention. Regarding the implementation timeline, the majority supported a one-month rollout, while a small proportion favoured a slightly longer timeframe, indicating a general preference for rapid implementation with limited variation in opinion. There was also a very strong consensus on leadership responsibility, with almost all experts supporting the oversight of the strategic plan by nursing managers, highlighting the central role of nursing leadership in driving HPV-related health education at the facility level.

#### 3.2.5. Thoroughly Elucidate the HPV Screening Procedure for the Patient and Encourage the Patient to Seek Clarification by Asking Questions

There was a strong overall consensus on the implementation of the strategic plan, with the majority (73.3%) of participants, expressing agreement and strong support. This reflects the broad acceptance of the proposed intervention among experts. Participants also highlighted the importance of developing procedural tools or information materials to support implementation. This indicates a shared view that nurses need structured guidance to effectively communicate HPV procedures to patients. Regarding the implementation timeline, responses varied, with preferences ranging from one to three months. This pattern suggests that there is no clear consensus on timing, although there is a general tendency toward short-term implementation. There was also strong agreement on leadership responsibility, with most participants supporting nursing managers as key drivers of implementation, indicating confidence in facility-level leadership for execution of the strategy.

#### 3.2.6. Providing Details on HPV Screening and Vaccination on Social Media Platforms

Strategy six focused on disseminating HPV information through social media platforms such as Facebook and WhatsApp to increase awareness of HPV, vaccination, and screening options. There was a strong consensus of 80.0% among participants supporting the implementation of this strategy, indicating high acceptance of digital platforms as an effective tool for health communication. Participants also stressed the importance of regularly updating social media content, highlighting that the effectiveness of this strategy depends on the relevance and timeliness of the information shared. On the other hand, no clear consensus emerged on the implementation timeline, as responses varied between different proposed periods, suggesting differing views on feasibility and urgency. However, there was strong agreement on governance, with most of the participants supporting the Department of Communication and Digital Technologies as the lead implementing authority, reflecting the need for structured coordination and digital expertise in the execution of the strategy. The panel of experts provided no additional input or recommendations.

#### 3.2.7. Implementation of a Module or Curriculum Covering Diseases with High Mortality Rates in Primary, Secondary, and Tertiary Education Institutions

The seventh strategy focused on integrating curriculum content on infections associated with high mortality rates at all educational levels. There was a strong general agreement (73.3%) among participants that supported the implementation of this strategy, indicating recognition of the importance of embedding health education within formal learning systems. Participants also supported the incorporation of this content into the broader educational curriculum, reflecting consensus on the need for structured and sustained education at all levels of education. On the other hand, no clear consensus emerged on the implementation timeline, as responses varied between periods of one, two, three, and six months. This pattern suggests different views on feasibility and readiness for implementation, although there is a general inclination toward shorter timeframes. However, there was strong agreement on institutional responsibility, and most participants supported the Department of Education being the lead implementing body, highlighting the importance of formal governance in curriculum integration. No additional suggestions or recommendations were provided by the expert panel.

#### 3.2.8. Organising an HPV Screening Workshop or Training Programme Tailored for Newly Graduated Professional Nurses

There was an overwhelming consensus among experts in support of the implementation of the strategic plan, indicating a very strong acceptance of the proposed intervention. Overall, 93.3% of the participants strongly agreed that workshops or training programmes should be developed for newly qualified nursing managers to support effective implementation, reflecting the perceived importance of capacity building for sustainability. On the other hand, no clear consensus emerged regarding the implementation timeline, with responses distributed across one-, two-, three-, and six-month options. This variation suggests different perceptions of feasibility and urgency, although preferences generally leaned toward short- to medium-term implementation. However, there was unanimous agreement on governance, with all participants supporting the National Department of Health as the responsible authority for implementation, highlighting the need for centralised oversight and coordination. The expert panel did not provide additional suggestions or recommendations.

### 3.3. Round Two: Strategies to Raise Awareness Among Women About HPV Screening and Vaccination in Limpopo Province

The second round focused on evaluating the effectiveness of strategies to inform women in Limpopo Province about HPV screening and vaccination. Compared to Round 1, increased agreement was observed, indicating convergence of expert opinion. Consensus was achieved during this phase. Table 3 presents the Round 2 strategies for raising awareness about HPV screening and vaccination among women.

#### 3.3.1. Informing Women About the Most Recent Updates in the HPV Screening Guideline

A consensus was achieved on the first strategy; there was strong overall agreement of 93.3% among respondents supporting the implementation of this strategy, indicating a high level of acceptance of its importance. Participants also reached consensus on the need to develop teaching programmes in healthcare facilities, reflecting agreement that structured educational support is essential for effective implementation. Regarding the timeline for implementation, consensus was reached on a one-month timeline, and most participants strongly supported rapid implementation, indicating agreement on the urgency of rolling out the strategy. There was also 100% unanimous agreement on responsibility, and all respondents indicated that registered nurses and nursing managers at the facility level should oversee implementation, highlighting strong confidence in clinical leadership for execution. Respondents did not provide additional input or recommendations.

#### 3.3.2. Conducting HPV Awareness Campaigns in Primary, Secondary and Tertiary Educational Institutions

A consensus was achieved on the second strategy; there was 100% unanimous support for the implementation of this strategy, indicating a strong shared commitment to strengthening HPV awareness across educational levels. Participants also reached consensus on the development of structured HPV awareness campaigns, reflecting the agreement that formalised programmes are necessary for effective implementation. Regarding the implementation timeline, consensus was reached at six months, with most respondents supporting this timeframe, indicating agreement on a more realistic and structured rollout period. There was also strong agreement on governance responsibility, with participants indicating that the National Department of Health should oversee implementation, highlighting the need for central coordination at the national level. Respondents provided no additional input or recommendations.

#### 3.3.3. Implementation of the HPV Programme on Community Radio and Television Channels

A consensus was achieved on the third strategy. There was unanimous support for the implementation of this strategy, indicating strong agreement on the value of mass media in promoting HPV awareness. Participants also reached 100% full consensus on the development of structured HPV broadcast programmes, confirming the necessity of formalised content for effective dissemination through radio and television channels. Regarding the implementation timeline, consensus was reached on a six-month period, with most participants strongly supporting this timeframe, indicating agreement on a structured and realistic rollout period. There was also unanimous agreement on governance responsibility, with all participants indicating that both the Department of Communication and Digital Technologies and the National Department of Health should oversee implementation, highlighting the importance of interdepartmental collaboration. The participants did not provide additional suggestions nor recommendations.

#### 3.3.4. Providing Daily Health Education in PHC Facilities Concerning HPV Screening and Vaccination

A consensus was achieved on the fourth strategy; there was unanimous support for the implementation of this strategy, indicating strong agreement on the importance of continuous patient education in PHC settings. Participants also reached 100% full consensus on the development of structured educational programmes, reflecting agreement that formalised teaching initiatives are necessary to support effective HPV awareness among women. Regarding the implementation timeline, a consensus was reached on a one-month period, with most participants strongly supporting rapid implementation, indicating agreement on the urgency of introducing the strategy. There was also unanimous agreement on responsibility, with all participants indicating that registered nurses and nursing managers at the facility level should oversee implementation, highlighting strong confidence in clinical leadership to execute the strategy. Participants did not provide additional input or recommendations.

#### 3.3.5. Explain the HPV Screening Procedure to the Patient and Encourage the Patient to Seek Clarification by Asking Questions

A consensus was reached on the fifth strategy, which focused on providing comprehensive explanations of the HPV screening process and encouraging patient participation through questions to enhance understanding. There was 100% unanimous agreement on the development of procedural tools or informational materials to support clear communication of the HPV screening process, indicating strong support for structured patient education resources. Participants also reached consensus on the implementation timeline, most supporting a one-month period, reflecting agreement on the urgency of introducing the strategy. There was also 100% unanimous agreement on responsibility, with all participants indicating that registered nurses and nursing managers should oversee implementation, highlighting confidence in facility-level clinical leadership. The participants did not provide additional input or recommendations.

#### 3.3.6. Providing Information on HPV Screening and Vaccination Available on Social Media Platforms

A consensus was reached on the sixth strategy, which focused on disseminating HPV screening and vaccination information through leading digital platforms for messaging and social interaction. There was 100% unanimous support for the implementation of this strategy, reflecting strong agreement on the use of digital media as a key communication tool for HPV awareness. The participants also reached full consensus on the need to update social media platforms, indicating agreement that digital systems must be optimised to ensure effective dissemination of HPV-related information. Regarding the implementation timeline, consensus was reached on a six-month period, with most participants supporting this timeframe, suggesting agreement on a structured and realistic rollout period. There was also 100% unanimous agreement on governance responsibility, with all participants indicating that the Department of Communication and Digital Technologies should oversee implementation, highlighting the importance of centralised coordination in digital health communication. The participants did not provide additional input or recommendations.

#### 3.3.7. Implementation of a Module or Curriculum That Covers Diseases with High Mortality Rates in Primary, Secondary, and Tertiary Education Institutions

A consensus was achieved on the seventh strategy, which focused on incorporating a curriculum module on diseases with high mortality rates, including HPV, at the primary to tertiary education levels. There was 100% unanimous support for the implementation of this strategy, reflecting a strong agreement on the importance of integrating HPV-related content into formal educational systems. The participants also reached full consensus on the need to review and update the curriculum, indicating the agreement that curriculum alignment is essential for effective implementation at all education levels. Regarding the implementation timeline, consensus was reached on a three-month period, and most participants supported this timeframe, suggesting agreement on a structured but achievable rollout period. There was also 100% unanimous agreement on governance responsibility, with all participants indicating that the Department of Education should oversee implementation, highlighting the importance of institutional leadership in curriculum reform.

#### 3.3.8. Organising an HPV Screening Workshop or Training Programme Tailored for Newly Graduated Professional Nurses

A consensus was achieved on the eighth strategy, which focused on ensuring the provision of annual HPV screening training and workshops for newly qualified professional nurses. There was 100% unanimous support for the implementation of this strategy, reflecting a strong agreement on the importance of continuous professional development in HPV screening. Participants also reached a full consensus on the need for ongoing training for registered nurses, indicating that regular participation in HPV-related workshops is essential for maintaining competency in screening practices. Regarding the timeline of implementation, consensus was reached on 6 months, suggesting agreement on a realistic timeframe to initiate structured training programmes. There was also unanimous agreement on governance responsibility, with all participants indicating that the National Department of Health should oversee implementation, highlighting the importance of national coordination in the sustainability of training initiatives. The participants provided no additional input or recommendations.

**KENDALL’S W**

Formula:

$$\chi^{2}=m(n-1)W$$

- ***m*** = 15 experts.
- ***n*** = 8 strategies.

$$\chi^{2}=15\times 7\times 0.00254\approx 0.27$$

***χ*^2^** ≈ 0.27 (not significant).

These results indicate virtually no measurable agreement. Kendall’s coefficient of concordance indicated a very low level of agreement between experts (*W* ≈ 0.003, **χ^2^** = 0.27, *p* > 0.05). However, this result is interpreted with caution due to the limited variability in responses, as most experts rated all items as ‘strongly agree’, resulting in restricted dispersion and reduced discriminatory power of the test. Although Kendall’s coefficient of concordance was not informative due to the limited variability of responses (ceiling effect), consensus was established based on high levels of agreement across experts, as indicated by uniformly high ratings (predominantly ‘agree’ and ‘strongly agree’), low spread of responses, and consistent endorsement of all proposed strategies.

Although all strategies reached a unanimous consensus among participants, a comparative analysis based on perceived importance, feasibility, and expected impact revealed meaningful distinctions. Strategies involving mass media and digital platforms (community radio, television, and social networks) were perceived to have the greatest potential impact, due to their wide population reach and ability to disseminate information rapidly. On the other hand, facility-based strategies, such as daily health education and patient–provider communication, were considered highly feasible and immediately implementable, as they rely on existing healthcare structures and personnel.

Educational and training-focused strategies, including curriculum integration and professional development for nurses, were considered to have a long-term impact, although their implementation may require more time and systemic coordination. Overall, the findings suggest that a multilevel approach, combining high-impact mass communication strategies with feasible facility-level interventions and long-term educational reforms, is most appropriate for improving HPV awareness and uptake.

## 4. Discussion

Although the strategies identified in this study align with established public health approaches recommended by the World Health Organisation and the South African National Department of Health, the key contribution of this study lies in its contextualization within a rural South African setting. These strategies were developed and refined through a Delphi process involving local healthcare professionals, ensuring that they are feasible and relevant to the specific challenges of Limpopo Province. Furthermore, this study addresses a critical literature gap, as existing frameworks often provide broad recommendations without clear guidance on context-specific implementation. By translating these recommendations into practical, prioritised strategies, this study contributes to bridging the gap between policy and practice. From an implementation perspective, the findings provide insight into feasible delivery approaches and responsible stakeholders, thus enhancing the potential for successful uptake of HPV screening and vaccination programmes.

The findings of this study are consistent with global and national frameworks for HPV prevention, including those proposed by the World Health Organisation and the South African National Department of Health, which emphasise the importance of vaccination, screening, and public awareness. However, while these frameworks provide a broad strategic direction, they often lack context-specific implementation guidance. This study extends these frameworks by offering locally relevant, expert-validated strategies tailored to the primary healthcare context in Limpopo province, thus enhancing their applicability in resource-constrained settings.

### 4.1. Informing Women About the Most Recent Updates in the HPV Screening Guideline

The objective of this strategy is to enhance women’s awareness of the updated HPV screening guidelines. This is essential, as HPV is the primary cause of cervical cancer and early detection significantly reduces the burden of the disease. From a Health Belief Model (HBM) perspective, improving awareness increases women’s perceived susceptibility to HPV infection and perceived severity of cervical cancer, which are key drivers of preventive health behaviour. Improved knowledge of screening guidelines also strengthens perceived benefits, as women are better able to understand the effectiveness of early detection and treatment in preventing complications. At the same time, providing clear information can help reduce perceived barriers, such as fear, uncertainty, or lack of understanding about the screening process.

As a result, informed women are more likely to perform timely screening, adhere to follow-up recommendations, and make confident health decisions. This ultimately supports sustained preventive behaviour and contributes to improved outcomes of cervical cancer. Furthermore, educated women are more likely to encourage others to undergo HPV screening, stating that HPV screening is the most effective method for early detection of HPV-related cancers, contributing to a significant decline in cancer rates in various regions of America [6,7,8].

### 4.2. Conducting HPV Awareness Campaigns in Primary, Secondary, and Tertiary Educational Institutions

The primary purpose of this strategy is to provide young people with comprehensive knowledge of HPV and its prevention. This is particularly important given that HPV is transmitted primarily through sexual contact, placing young people at increased risk of infection. From an HBM perspective, targeted awareness campaigns aim to enhance perceived susceptibility among young people by increasing their understanding of personal risk. Additionally, such campaigns strengthen perceived severity by highlighting the potential long-term consequences of HPV infection, including cervical cancer. At the same time, they improve the perceived benefits of preventive behaviours such as vaccination and early detection. HPV awareness campaigns have therefore been identified as critical in improving knowledge and promoting informed decision-making regarding prevention and risk reduction among young populations. These campaigns could include in-person visits to schools, where students would receive detailed information about HPV, its infections, screening methods, and vaccination. Such sessions would also allow children to ask questions, improving their overall understanding of HPV [9,10,11]. Health awareness initiatives can help reduce the stigma associated with HPV by educating the public on its prevalence and associated risks, as well as preventive measures. Furthermore, HPV awareness campaigns can make the virus easier to understand and promote open conversations, creating a more comfortable environment to discuss the topic [12,13].

### 4.3. Implementation of the HPV Programme on Community Radio and Television Channels

The objective of this strategy is to promote awareness of HPV through broadcast media as a cost-effective method to reach a wide and diverse population. Radio and television are highly effective communication tools for public health messaging due to their accessibility across different age, gender, and socioeconomic groups. Their wide reach makes them particularly suitable for disseminating HPV-related information in communities where access to printed or digital health resources may be limited.

From an implementation perspective, these platforms also offer a cost-effective alternative to other forms of health communication, enhancing their suitability for resource-constrained settings. Increased exposure to HPV information through media channels can improve community knowledge and may indirectly influence family-level awareness, as informed parents are more likely to guide and encourage adolescents to engage with preventive health messages.

In particular, radio remains an important source of information for older and less literate populations, while television effectively engages younger audiences. This dual reach improves the potential impact of media-based interventions in improving HPV awareness across generations. It was stressed that parents’ understanding of HPV is vital. Informed parents are likely to share this knowledge with household members and ensure that those eligible receive the recommended vaccine doses [14,15].

### 4.4. Providing Daily Health Education in PHC Facilities Concerning HPV Screening and Vaccination

The objective of this strategy is to provide women with complete information regarding HPV testing procedures and vaccine opportunities available at healthcare facilities. Providing clear and structured health education is an important mechanism to improve awareness of HPV and promote informed health decisions. Interactions with healthcare providers can further strengthen understanding, as women can receive personalised information and clarification, which enhances trust and knowledge retention. From a behavioural perspective, informed women are more likely to participate in preventive health actions, including adherence to recommended screening intervals and attendance at follow-up appointments. An increased understanding of HPV, its high-risk strains, and associated health consequences also supports improved awareness of risk and proactive health-seeking behaviour. Furthermore, health education on HPV vaccination improves knowledge of vaccine types, eligibility criteria, and appropriate vaccination schedules within the South African context. This knowledge may extend beyond individual behaviour, as informed women, particularly mothers, are more likely to influence family health decisions by encouraging vaccination among their children, thus contributing to broader community-level prevention efforts. A study was conducted and stressed that health education on HPV and vaccination is crucial, as it can increase vaccination uptake and contribute significantly to reducing HPV incidence [16,17].

### 4.5. Thoroughly Elucidate the HPV Screening Procedure to the Patient and Encourage the Patient to Seek Clarification by Asking Questions

The objective of this strategy is to ensure that women understand the HPV screening procedure and could clarify any uncertainties. Providing clear and comprehensive explanations before screening can reduce anxiety and improve adherence to recommended screening guidelines. Effective communication between nurses and patients is essential to building trust, which in turn strengthens the patient–provider relationship and increases the likelihood of compliance with clinical recommendations. From a behavioural perspective, patients are more likely to trust healthcare professionals who demonstrate competence and provide clear information, enhancing the acceptance of preventive procedures. Understanding the screening process may also reduce fear and psychological distress by allowing patients to anticipate what to expect during the procedure. Additionally, a detailed explanation supports informed consent, ensuring that patients fully understand the risks, benefits, and purpose of the procedure before agreeing to participate. This process promotes patient autonomy and reinforces ethical clinical practice. Allowing patients to ask questions further enhances comprehension, improves information retention, and supports adherence to follow-up appointments, thus contributing to continuity of care. It was indicated that despite receiving information during the invitation to HPV screening, some patients still have questions about HPV [18,19]. This underscores the importance of thoroughly explaining the HPV testing procedure to patients who choose to undergo screening and ensuring that they can address any uncertainties they may have.

### 4.6. Providing Information on HPV Screening and Vaccination on Social Media Platforms

The objective of this strategy is to reach women and young people through digital platforms, recognising their widespread use for communication, information sharing, and social interaction. Social media platforms such as WhatsApp and Facebook are particularly popular among adolescents and young adults, making them effective channels for disseminating health information and promoting engagement on sensitive topics such as HPV. From an implementation perspective, these platforms offer an accessible and less intimidating alternative to face-to-face communication, allowing users to engage with health information at their own pace. This familiarity with digital interaction can enhance receptiveness to HPV-related messages and improve information uptake. The proposed use of a structured digital communication channel, such as a dedicated WhatsApp contact, further enhances accessibility by enabling users to access customised HPV information, including definitions, screening procedures, types of infections, and vaccination options. This interactive and user-driven approach supports personalised learning, which may improve understanding, trust in information, and willingness to adopt preventive behaviours. According to a study conducted on “improving HPV vaccination rates among young adolescents through health education”, it was found that awareness campaigns conducted on social media platforms are more effective than those using brochures and printed materials [9,10,11].

### 4.7. Implementation of a Module or Curriculum That Covers Diseases with High Mortality Rates in Primary, Secondary, and Tertiary Education Institutions

The objective of this strategy is to integrate HPV education into the school curriculum, targeting learners at an early age to promote awareness of the importance of preventing HPV-related infections. Introducing HPV-related content within formal education systems plays a critical role in improving vaccine coverage and addressing misconceptions associated with the virus and its vaccine. From an HBM perspective, early education improves the perceived susceptibility by increasing awareness that HPV can affect sexually active individuals, even at a young age. It also strengthens perceived severity by highlighting the potential long-term consequences of infection, including cervical cancer. Furthermore, curriculum-based education improves the perceived benefits of vaccination and screening by clarifying their protective value, while helping to reduce perceived barriers such as misinformation, fear, and stigma. Well-informed students are more likely to adopt preventive health behaviours and can also act as secondary sources of information by sharing knowledge within their families and communities. This contributes to greater dissemination of accurate information, in turn reducing misconceptions about HPV and vaccination, and fosters a more supportive environment for preventive health decision-making. A study conducted at the University of South Carolina highlights the significant role that college and university health services play in promoting sexual health. It emphasises the importance of these services in the implementation of catch-up vaccination programmes for young adults who are not vaccinated or under-vaccinated against HPV [20,21]. The study underscores the need for greater efforts to raise awareness about HPV and to improve vaccination rates, particularly in the southern region of the United States.

### 4.8. Organising an HPV Screening Workshop or Training Programme Tailored for Newly Graduated Professional Nurses

The objective of this strategy is to enhance nurses’ knowledge and competence in conducting HPV screening procedures and to ensure continuous familiarity with updated policies and guidelines. This is essential to maintain high-quality patient care and ensure standardised practice in cervical cancer prevention services. Structured training for newly qualified registered nurses is particularly important, as undergraduate clinical exposure is often limited, and students may not have enough opportunities to develop and consolidate practical screening skills before entering clinical practice. This skill gap can affect confidence and competency in delivering HPV-related services. From an implementation perspective, workshops and in-service training programmes offered by healthcare institutions provide a mechanism to bridge this gap by strengthening clinical competence, improving adherence to guidelines, and ensuring consistency in service delivery. Such training initiatives therefore support both professional development and the overall quality of HPV screening services within primary healthcare settings. Workshops and training programmes specifically targeting HPV screening were highlighted to improve the diagnostic skills of healthcare workers. The study proposes that regular implementation of such workshops would be beneficial in maintaining and further improving diagnostic proficiency among healthcare workers [22,23].

## 5. Study Limitations

This study has several limitations that must be acknowledged. Although participants completed the Delphi questionnaires individually, data collection occurred within a shared environment, which may have influenced responses due to the potential for perceived lack of anonymity. However, measures were taken to ensure that responses remained independent and were not discussed among participants during completion. Second, the relatively small and context-specific sample of experts can limit the generalizability of the findings beyond similar healthcare settings. Third, while consensus was achieved using predefined criteria, the results reflect expert opinion rather than empirical evidence of the effectiveness of the proposed strategies. Additionally, reliance on self-reported data may introduce response bias. Despite these limitations, the study provides valuable information on context-specific strategies to improve HPV awareness, screening, and vaccination, and offers a foundation for future research and implementation.

## 6. Conclusions

Insufficient knowledge of human papillomavirus contributes to low awareness of HPV screening and vaccination, which is concerning given that early detection and treatment can prevent serious health outcomes, including cervical cancer. This study identified critical gaps in HPV awareness among women and healthcare providers in South Africa, highlighting the need for targeted interventions to strengthen prevention efforts. The E-Delphi process confirmed the effectiveness of proposed strategies, with strong expert agreement that these approaches could enhance awareness, reduce vaccine hesitancy, and improve screening uptake.

These findings have important practical implications for policymakers and primary healthcare managers. The identified strategies can be used to inform the design and implementation of targeted health education programmes, strengthen community outreach initiatives, and support the integration of HPV awareness into routine PHC services in Limpopo Province and similar settings.

## Figures and Tables

**Table 1:** Summary of Socio-demographic data.

Demographic Characteristics	Frequency	Percentage
Gender		
Female	(12)	(80%)
Males	(3)	(20%)
Other	(0)	(0)
Total	(15)	(100%)
Experiences in practice		
5–9 years	(2)	(13.3%)
10–14 years	(7)	(46.7%)
15 years and older	(6)	(40.0%)
Total	(15)	(100%)
Profession		
Experienced nursing managers	(4)	(26.7%)
Nursing managers with a speciality in PHC	(9)	(60.0%)
Nursing managers with an obstetrics and gynaecology speciality	(2)	(13.3%)
Total	(15)	(100%)

**Table 2:** Round One: Strategies to raise awareness among women towards HPV screening and vaccination in Limpopo province. A = agree, U = Uncertain, D = Disagree, SD = strongly disagree.

Strategies	A	%	SA	%	U	%	D	%	SD	%
1. Informing women about the latest updates in the human papillomavirus screening guideline.	7	46.7	8	53.3	0	0	0	0	0	0
1.1. Action: Establish an educational curriculum in primary health care centres.	8	53.3	7	46.7	0	0	0	0	0	0
1.2. Person in charge: RNs and facility nursing managers	8	53.3	7	46.7	0	0	0	0	0	0
2. Conducting human papillomavirus awareness campaigns in primary, secondary and tertiary educational institutions.	11	73.3	4	26.7	0	0	0	0	0	0
2.1. Action: create a programme for HPV awareness campaign	10	66.7	5	33.3	0	0	0	0	0	0
2.2. Person in charge: NDoH	12	80.0	3	20.0	0	0	0	0	0	0
3. Implementation of the human papilloma virus programme on community radio and television channels	12	80.0	3	20.0	0	0	0	0	0	0
3.1. Action: create an HPV programme for broadcast on radios and televisions	10	66.7	5	33.3	0	0	0	0	0	0
3.2. Person in charge: DOCDT and NDoH	12	80.0	3	20.0	0	0	0	0	0	0
4. Providing daily health education at primary health care facilities concerning human papillomavirus screening and vaccination	11	73.3	4	26.7	0	0	0	0	0	0
4.1. Action: create a health education programme to inform women about HPV in primary health care centres.	12	80.0	3	20.0	0	0	0	0	0	0
4.2. Person in charge: RNs and facilities nursing managers	14	93.3	1	6.7	0	0	0	0	0	0
5. Thoroughly elucidate the HPV screening procedure for the patient and encourage the patient to seek clarification by asking questions	11	73.3	4	26.7	0	0	0	0	0	0
5.1. Action: Create an informative tool or letter outlining the HPV screening procedure of HPV screening	11	73.3	4	26.7	0	0	0	0	0	0
5.2. Person in charge: RNs and Facilities nursing managers	12	80.0	3	20.0	0	0	0	0	0	0
6. Making information on HPV screening and vaccination available on social media platforms	12	80	3	20.0	0	0	0	0	0	0
6.1. Action: Action: Upgrade social media software to disseminate information on HPV	12	80.0	3	20.0	0	0	0	0	0	0
6.2. Person in charge: DOCDT	12	80.0	3	20.0	0	0	0	0	0	0
7. Implementation of a module or curriculum covering diseases with high mortality rates in primary, secondary, and tertiary education institutions	11	73.3	4	26.7	0	0	0	0	0	0
7.1. Action: Revise the educational curriculum across all academic levels from primary to tertiary	11	73.3	4	26.7	0	0	0	0	0	0
7.2. Person in charge: Department of education	12	80.0	3	20.0	0	0	0	0	0	0
8. Organisation of a Human Papilloma Virus screening workshop or training programme tailored for recently graduated professional nurses	14	93.3	1	6.7	0	0	0	0	0	0
8.1. Action: Establish an HPV screening workshop/training programme tailored for newly qualified professional nurses	14	93.3	1	6.7	0	0	0	0	0	0
8.2. Person in charge: NDOH	15	100	0	0	0	0	0	0	0	0

**Table 3:** Round Two: Strategies to raise awareness among women towards HPV screening and vaccination in Limpopo province. A = agree, U = Uncertain, D = Disagree, SD = strongly disagree.

Strategies	A	%	SA	%	U	%	D	%	SD	%
1. Informing women about the latest updates in the Human Papillomavirus screening guideline	1	6.7	14	93.3	0	0	0	0	0	0
1.1. Action: Establish an educational curriculum in primary health care centres	1	6.7	14	93.3	0	0	0	0	0	0
1.2. Person in charge: RNs and facility nursing managers	0	0	15	100	0	0	0	0	0	0
2. Conducting human papillomavirus awareness campaigns in primary, secondary and tertiary educational institutions	1	6.7	14	93.3	0	0	0	0	0	0
2.1. Action: create a programme for HPV awareness campaign	1	6.7	14	93.3	0	0	0	0	0	0
2.2. Person in charge: NDoH	1	6.7	14	93.3	0	0	0	0	0	0
3. Implementation of the Human Papillomavirus programme on community radio and television channels	0	0	15	100	0	0	0	0	0	0
3.1. Action: create an HPV programme for broadcast on radios and televisions	0	0	15	100	0	0	0	0	0	0
3.2. Person in charge: DOCDT and NDoH	0	0	15	100	0	0	0	0	0	0
4. Provide daily health education at primary health care facilities on Human Papillomavirus screening and vaccination	0	0	15	100	0	0	0	0	0	0
4.1. Action: Create a health education programme to inform women about HPV in primary health care centres	0	0	15	100	0	0	0	0	0	0
4.2. Person in charge: RNs and the facility’s nursing manager	0	0	15	100	0	0	0	0	0	0
5. Clearly explain the HPV screening procedure to the patient and encourage her to seek clarification by asking questions	0	0	15	100	0	0	0	0	0	0
5.1. Action: create an informative tool or letter outlining the HPV screening procedure of HPV screening	0	0	15	100	0	0	0	0	0	0
5.2. Person in charge: RNs and Facilities nursing managers	0	0	15	100	0	0	0	0	0	0
6. Making information on HPV screening and vaccination available on social media platforms	0	0	15	100	0	0	0	0	0	0
6.1. Action: Upgrade social media software to disseminate information on HPV	0	0	15	100	0	0	0	0	0	0
6.2. Person in charge: DOCDT	0	0	15	100	0	0	0	0	0	0
7. Implementation of a module or curriculum covering diseases with high mortality rates in primary, secondary, and tertiary education institutions	0	0	15	100	0	0	0	0	0	0
7.1. Action: Revise the educational curriculum across all academic levels from primary to tertiary	0	0	15	100	0	0	0	0	0	0
7.2. Person in charge: Department of Education	0	0	15	100	0	0	0	0	0	0
8. Organisation of a Human Papillomavirus screening workshop or training programme tailored for recently graduated professional nurses	0	0	15	100	0	0	0	0	0	0
8.1. Action: Establish an HPV screening workshop/training programme tailored for newly graduated professional nurses	0	0	15	100	0	0	0	0	0	0
8.2. Person in charge: NDoH	0	0	15	100	0	0	0	0	0	0

## Data Availability

The original contributions presented in this study are included in the article. Further inquiries can be directed to the corresponding author. AI Disclosure Statement: Artificial intelligence tools (ChatGPT) version: 5.3 were used solely for language editing, paraphrasing, and improving the grammar and clarity of the manuscript. The tool assisted in refining sentence structure and enhancing academic writing quality without altering the scientific content. It was not used for data collection, data analysis, interpretation of results, or generation of findings, strategies, or conclusions. All methodological decisions, analysis, and interpretation were independently conducted by the authors, and the final manuscript was critically reviewed and validated to ensure accuracy and academic integrity.

## Abbreviations

List of Abbreviations Used: HPV—human papilloma virus, RN—registered nurses, DOCDT—department of communication and digital technology, NDOH—national department of health, PHC—primary health centre, PHC—primary health care.

## Appendix A. Delphi Technique

**RESEARCH QUESTIONNAIRE**
**Study Title**: Strategies to create an awareness regarding human papillomavirus screening and screening among women in Limpopo Province, South Africa
**Code**
**Instructions**

1. Answer all questions in this questionnaire.
2. Tick your answer in the space provided.
3. Do not tear any page.
4. Do not write your name.

**Table A1:** **Section A: Demographic data of the respondents.**

Question No.	Questions	Answers	Fill in the Space Provided
1.	Gender	a. Female	
		b. Male	
		c. Other	
2.	Experience in practice	a. 5–9 years	
		b. 10–14 years	
		c. 15 and above	
3.	Profession	a. Experienced professional nurse	
		b. Nurse with PHC speciality	
		c. Professional nurse with obstetrics and gynaecology speciality	
		d. Other	

**Table A2:** **Section B: Strategies to create an awareness regarding human papillomavirus screening (tick the appropriate response).**

Strategy No.	Strategies	Agree	Strongly Agree	Uncertain	Disagree	Strongly Disagree
4.	Informing women about the most recent updates in the Human Papillomavirus screening guidelines					
4.1.	Action: Establish an educational curriculum at primary health care centres					
4.2.	timeline:					
4.2.1.	1 Month					
4.2.2.	2 Months					
4.2.3.	3 Months					
4.2.4.	6 Months					
4.3.	Person in charge: RNs and facility nursing managers					
Inputs/recommendations						
5.	Conducting Human Papillomavirus awareness campaigns in primary, secondary, and tertiary educational institutions					
5.1.	Action: Create a programme for HPV awareness campaigns					
5.2.	Timeline:					
5.2.1.	1 Month					
5.2.2.	2 Months					
5.2.3.	3 Months					
5.2.4.	6 Months					
5.3.	Person in charge: National Department of Health (NDoH)					
Inputs/recommendations						
6.	Implementation of the Human Papillomavirus program on community radio and television channels					
6.1.	Action: Create a HPV programme for broadcasting on radios and televisions					
6.2.	Timeline:					
6.2.1.	1 Month					
6.2.2.	2 Months					
6.3.3.	3 Months					
6.3.4.	6 Months					
6.3.	Person in charge: Department of Communications and NDoH					
Inputs/recommendations						
7.	Providing daily health education at PHC facilities concerning HPV screening and vaccination					
7.1.	Action: Create a health education programme for informing women about HPV at primary health care centres					
7.2.	Timeline:					
7.2.1.	1 Month					
7.2.2.	2 Months					
7.3.3.	3 Months					
7.3.4.	4 Months					
7.3.	Person in charge: RNs and facility nursing managers					
Inputs/recommendations						
8.	Thoroughly elucidate the HPV screening procedure to the patient and encourage her to seek clarification by asking questions					
8.1.	Action: Create an informative tool or letter outlining the procedure of HPV screening					
8.2.	Timeline:					
8.2.1.	1 Month					
8.2.2.	2 Months					
8.2.3.	3 Months					
8.2.4.	6 Months					
8.3.	Person in charge: RNs and facility nursing managers					
Inputs/recommendations						
9.	Making information about HPV screening and vaccination available on social media platforms					
9.1.	Action: Upgrade social media software to disseminate information on HPV					
9.2.	Timeline:					
9.2.1.	1 Month					
9.2.2.	2 Months					
9.2.3.	3 Months					
9.2.4.	6 Months					
9.3.	Person in charge: Department of Communication and Technology Digital					
Inputs/recommendations						
10.	Implementation of a module or curriculum covering diseases with high mortality rates in primary, secondary, and tertiary education institutions					
10.1.	Action: Revise the educational curriculum across all academic levels from primary to tertiary					
10.2.	Timeline:					
10.2.1.	1 Month					
10.2.2.	2 Months					
10.2.3.	3 Months					
10.2.4.	6 Months					
10.3.	Person in charge: Department of Education					
Inputs/recommendations						
11.	Organising a Human Papillomavirus screening workshop or training programme tailored for recently graduated professional nurses					
11.1.	Action: Establish a HPV screening workshop/training programme tailored for newly graduated professional nurses					
11.2.	Timeline:					
11.2.1.	1 Month					
11.2.2.	2 Months					
11.2.3.	3 Months					
11.2.4.	6 Months					
11.3.	Person: National Department of Health					
